# Whole blood donor deferral analysis at a center in Western India

**DOI:** 10.4103/0973-6247.67035

**Published:** 2010-07

**Authors:** Naveen Agnihotri

**Affiliations:** *Department of Transfusion Medicine, Blood Bank, Aditya Birla Memorial Hospital, Chinchwad, Pune- 411 033, Maharashtra, India*

**Keywords:** Anemia in blood donors, blood donor deferral, deferral criteria, deferral reasons, donor rejection, hypertension in blood donors, medication history in blood donors

## Abstract

**Introduction:**

Deferrals lead to loss of precious whole blood donors (WBD) and blood units available for transfusion purposes. Knowledge of rate and causes of donor deferral can guide the recruitment strategy for WBD.

**Aim:**

To find the incidence and causes of deferral in Indian WBD and apply relevant findings to modify recruitment strategy for blood donors.

**Materials and Methods:**

Data for WBD presenting for donation in a blood center and outdoor camps over one and half year were analyzed retrospectively. National guidelines were used for selection and deferral of WBD.

**Result:**

736 (11.6%) WBD were deferred out of 6357 presenting for donation during the study period. Most (69.8%) of the donors were deferred on physical examination and hemoglobin (Hb) testing. Most common reasons for deferral were low Hb (55.8%), abnormal blood pressure (11.1%), medication (6.9%) and underweight donors (2.9%). Significantly more volunteers were deferred than relative donors (13.97% vs 5.80%; *P*<0.000). Females were found to have higher deferral rate than males (53.5% vs 6.9%; P=0.000) and higher odds ratio for deferral (15.4). Donors older than 40 years of age had significantly higher chance of being deferred (*P*<0.05).

**Discussion and Conclusion:**

It is important to determine the rate and causes of WBD deferral to guide the recruitment and retention efforts at local, regional, and national level.

## Introduction

Blood donor deferral is a painful and sad experience for the blood donor as well as the blood center screening the donor. These deferrals “bleed” the donor-recruiting efforts of a blood center, necessitating more efforts diverted to new recruitments. Moreover, deferring prospective donors often leaves them with negative feelings about themselves as well as the blood donation process.[1] Additionally these donors are less likely to return for blood donation in future.[2] Nonetheless, criteria for these deferrals and their implementation strongly influence the quality of blood supply in a population. Thus, every blood centre has to balance the fulcrum between acceptable quality and desired quantity.

There is dearth of indigenous studies (done and/or published) and “informed medical opinion” on whole blood donor deferrals in India. Lack of “sensitization” to these deferral criteria seems to be both cause and effect of this “scarcity of literature” on the topic.

Nodal agencies like the National AIDS Control Organization (NACO) and the State Blood Transfusion Councils (SBTCs) do not actively collect data on donor deferrals. Their formats for data collection are more inclined toward “quantity” of supply and deferrals due solely to infectious marker positivity in donated units. As a result most of the efforts at government, community, and individual level are focused at recruiting more and more new donors while ignoring the retention and re-entry of those recruited but deferred due to various causes. This can be achieved by analyzing the reason of these deferrals amongst blood donors, addressing the issue and ameliorating the cause if possible.

The criteria for prospective blood donor selection and deferral in India are provided by the Drugs and Cosmetic Act 1940 (and rules thereunder) supplemented by the Technical Manual (Directorate General of Health Services, MOH and FW, Govt. of India).

The few studies done in India in the past have provided different common reasons for deferral of whole blood donors, highlighting differing demographic profile in different parts of the country.[3,4] The present study was undertaken to analyze the deferral incidence and pattern among blood donors in an Indian Blood Center with an objective to review the Center”s policy for recruitment and retention of blood donors.

## Materials and Methods

Data were analyzed retrospectively for whole blood donations over a period of one and half years, from January 2008 to June 2009. Donors presenting at indoor as well as outdoor locations were included in the study. Standard Operating Procedures based on national guidelines were used for donor selection and deferral. Briefly, the cutoff for hemoglobin (Hb) was 12.5 gm/dl by the finger prick method; all male donors were screened for Hb using CuSO_4_ and all female and doubtful values for male donors (on CuSO_4_) were confirmed for correct Hb by Hemocue Hb 201+ (HemoCue AB, Angelholm, Sweden). Using these two time tested methods for Hb estimation together as complimentary tests has proved to be a cost-effective and sensitive screening test, as has been proved in Indian scenario also.

Donors with systolic blood pressure (B.P. as measured by fully automatic blood pressure monitor Accumam, Morepen Laboratories) between 100 and 180 mmHg and diastolic blood pressure between 50 and 100 mmHg alone were accepted for blood donation; an average of three measurements of B.P. was taken for those not falling within this range for systolic and/or diastolic B.P.

Deferral reasons were analyzed amongst Relative-Voluntary, male-female and various age group categories. Donors were categorized into five conventional age group categories for the sake of convenience and analysis.

Related donors were not labeled as “replacement” as there was no compulsion to donate blood and desired blood component(s) was almost always issued to the patient before any related donors were requested to donate blood. Thus related donors were not replacement donors in conventional sense and were under no obligation to replace blood supplied to their patient.

First time and repeat donors were not segregated and for the sake of simplicity of analysis, all repeat presentations were considered as independent attempts for blood donation.

First analysis of data focused on descriptive statistics of all variables. Qualitative data were summarized as number of cases and percentage while quantitative data were presented as number of cases, mean, median, range, standard deviation, and 95% confidence interval for the difference. The significance limit was set at 0.05 and chi-square test was used to determine statistical significance. Odds ratio was calculated to determine the probability of deferral among male and female donors.

## Results

Total number of attempts for whole blood donations during the study period were 6357, where 4494 (70.7%) were volunteers and 1863 (29.3%) were related donors. Significantly more female donors presented as voluntary donors than related donors (12.4% voluntary vs 4.1% relative; *P*=0.000, Table 1).

**Table 1:** Demographic profile of donor population

Donor category	Male (%)	Female (%)	Total (%)
Related	1787 (28.1)	76 (1.2)	1863 (29.3)
Voluntary	3936 (61.9)	558 (8.8)	4494 (70.7)
Total	5723 (90.0)	634 (10.0)	6357 (100)

Of the total donors who presented for blood donation, 10% were females (n=634), however majority of them were deferred so that they contributed only 5.2% of selected donors [Table 2].

**Table 2:** Demographic profile of deferred donors

Donor category	Male (%)	Female (%)	Total (%)
Related	79 (10.7)	29 (4.0)	108 (14.7)
Voluntary	318 (43.2)	310 (42.1)	628 (85.3)
Total	397 (53.9)	339 (46.1)	736 (100)

Majority (57.7%) of the donors presenting for the donation were between 25 and 39 years of age [Table 3]

**Table 3:** Donor distribution according to age group

Age group (in years)	Deferred % (n)	Selected % (n)	Total % (n)	Deferral % in respective age category
<18	1.9 (14)	0 (0)	0.2 (14)	NA
18-24	22.7 (167)	25.6 (1441)	25.3 (1608)	10.4
25-39	48.9 (360)	58.9 (3308)	57.7 (3668)	9.8
40-54	21.2 (156)	13.9 (784)	14.8 (940)	16.6
>55	5.3 (39)	1.6 (88)	2.0 (127)	30.7
Total	100 (736)	100 (5621)	100 (6357)	

Total no of deferrals were 736 giving an overall incidence of 11.6%. Out of these 736 deferrals, 628 (85.3%) were volunteers and 108 (14.7%) were related donors. Common deferral reasons are tabulated in Table 4 according to the percentage of donors deferred for a particular reason.

**Table 4:** Deferral reasons in blood donors

Deferral Reason		% of Deferred Donors (n = 736)	% of Total Donors (n = 6357)
Physical Examination (69.8% of total deferrals)	Low Hemoglobin	55.8	6.5
	Abnormal Blood pressure	11.1	1.3
	Underweight	2.9	0.3
Medical history and interview (30.2% of total deferrals)	Antibiotics and other medications	6.9	0.8
	Menstrual cycle	2.7	0.3
	Age – over or under	2.7	0.3
	Previous donation within 3 months	2.0	0.2
	Surgical cause*	1.4	0.2
	H/o Jaundice/ Hepatitis B/ C	1.7	0.1
	Others†	12.8	1.6
	Total	100	11.6

* These were either due to past surgery within 3 months or scheduled for surgery shortly;

† Positive response to organ system disease warranting deferral, high risk donor, infection, under influence of alcohol, vaccination, breastfeeding, etc.

A majority (70%) of donors were deferred on physical examination during the donor selection process and most common reason (55.8%) for deferral was low hemoglobin. The next common reasons for deferral in our donor population were an abnormal blood pressure recording (either high or low) and ongoing medications (11.1% and 6.9%, respectively).

### Deferral reason comparison

Deferral reasons were compared among voluntary and relative donors [Table 5], male and female donors [Table 6] and across various age group categories [Table 7].

**Table 5:** Deferral reason comparison in voluntary and relative blood donors

	Deferral Reason	% (n) of Deferred relative donors	% (n) of Deferred voluntary donors
Deferral on physical examination	Low Hemoglobin	58.3 (63)*	55.4 (348)*
	Abnormal Blood pressure	15.7 (17)†	10.3 (65)†
	Underweight	3.7 (4)‡	2.7 (17)
Deferral on medical history and interview	Antibiotics and other medications	1.9 (2)	7.8 (49)‡
	Menstrual cycle	1.9(2)	2.9 (18)
	Age – over or under	0(0)	3.2 (20)
	Donation within 3 months	1.9 (2)	2.1(13)
	Surgical cause§	1.9 (2)	1.3 (8)
	H/o Jaundice/ Hepatitis B/ C	0.9(1)	1.9 (12)
	Others║	13.8 (15)	12.4 (78)
	Total	100 (108)	100 (628)

* most common cause;

† 2^nd^ most common cause

‡ 3^rd^ most common cause of deferral;

§ these were either due to past surgery within 3 months or scheduled surgery shortly;

║ Positive response to organ system disease warranting deferral, high risk donor, infection, under influence of alcohol, vaccination, baby feeding, history of dog bite, etc.

**Table 6:** Deferral reason comparison in female and male blood donors

Deferral Reason	% (n) of Deferred female donors	% (n) of Deferred male donors
Low Hemoglobin	77.9 (264)*	37.0 (147)*
Abnormal Blood pressure	2.7 (9)	18.4 (73)†
Underweight	3.5 (12)‡	2.3 (9)
Antibiotics and other medications	1.5 (5)	11.6 (46)‡
Menstrual cycle	5.3 (18)†	NA
Age – over or under	0.9 (3)	4.3 (17)
Donation within 3 months	0.6 (2)	3.3 (13)
Surgical cause§	0.6 (2)	2.0 (8)
H/o Jaundice/ Hepatitis B/ C	0.3 (1)	3.0 (12)
Others║	6.7 (23)	18.1 (72)
Total	100 (339)	100 (397)

* - most common cause;

† - 2^nd^ most common cause

‡ 3^rd^ most common cause of deferral;

§ these were either due to past surgery within 3 months or scheduled surgery shortly;

║ Positive response to organ system disease warranting deferral, high risk donor, infection, under influence of alcohol, vaccination, baby feeding, history of dog bite, etc.

**Table 7:** Deferral reason comparison in different age group blood donors

	% (n) of Deferred Donors*			
Deferral Reason	18-24 yrs	25-39 yrs	40-54 yrs	> 55 yrs
Low Hemoglobin	58.1 (97)†	57.8 (208)†	57.1 (89)†	43.6 (17)†
Abnormal Blood pressure	4.2 (7)§	8.3 (30)§	22.4 (35)‡	25.6 (10)‡
Underweight	8.4 (14)‡	1.7(6)	0.6 (1)	0(0)
Antibiotics and other medications	3(5)	9.2 (33)‡	7.1 (11)§	5.1 (2)
Menstrual cycle	4.2 (7)^c^	3.1 (11)	1.3 (2)	0(0)
Over-age	NA	NA	NA	15.4 (6)§
Donation within 3 months	2.4 (4)	3.1 (11)	0(0)	0(0)
Surgical cause ║	0.6(1)	1.1 (4)	2.5 (4)	2.6(1)
H/o Jaundice/ Hepatitis B/ C	2.4 (4)	2.5 (9)	0(0)	0(0)
Others **	16.7 (28)	13.2 (48)	9.0 (14)	7.7 (3)
Total	100 (167)	100 (360)	100 (156)	100 (39)

* Category with age less than 18 years not included;

† most common cause

§ 3^rd^ most common cause of deferral;

‡ 2^nd^ most common cause,

║ these were either due to past surgery within 3 months or scheduled surgery shortly;

** Positive response to organ system disease warranting deferral, high risk donor, infection, under influence of alcohol, vaccination, baby feeding, history of dog bite, etc.

**Figure 1:**
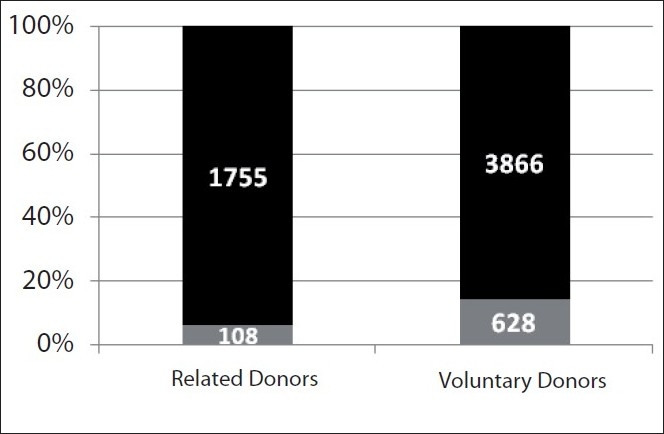
Percentage deferral in related and voluntary donors.

Significantly more volunteers were deferred as compared to related donors (13.97% vs 5.80%; *P*<0.000, Table 2 and Figure 1). Although, there was no significant difference in deferral due to two most common reasons, low Hb and abnormal B.P., in both the groups (P=0.65 and 0.14 for difference in deferral due to Hb and B.P., respectively), significantly more volunteers were deferred due to ongoing or past history of medication (7.8% in VD vs 1.9% in RD; *P*=0.04).

This difference was independent of deferral of female donors in both categories as deferral percentage in voluntary male donors was higher than related male donors (8.1% vs 4.4%, *P*<0.001; Table 2).

**Figure 2:**
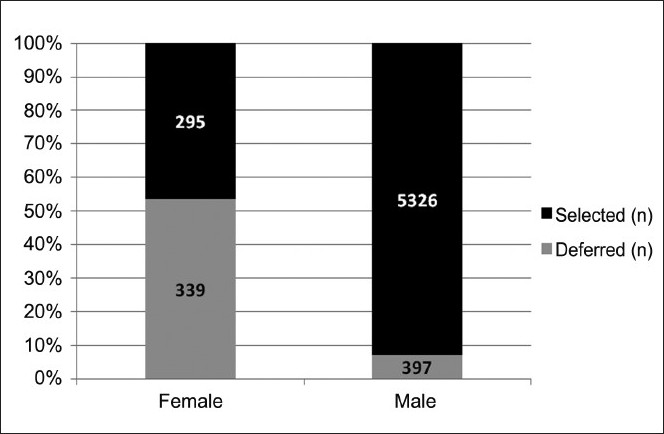
Deferral percentage in female and male donors.

Significantly more female donors were deferred as compared to male donors (53.5% vs 6.9%; *P*=0.000, Tables 2 and 6, Figure 2). Although the most common reason for deferral in both the genders was a low Hb, significantly higher number of females were deferred due to this reason alone (77.9% in females vs 37.0% in males; *P*=0.000). Odds ratio for deferral in female donors was 15.4 (95% confidence interval 12.8 – 18.6), implying thereby that chance of deferral in females is nearly 15 times higher as compared to males.

**Figure 3:**
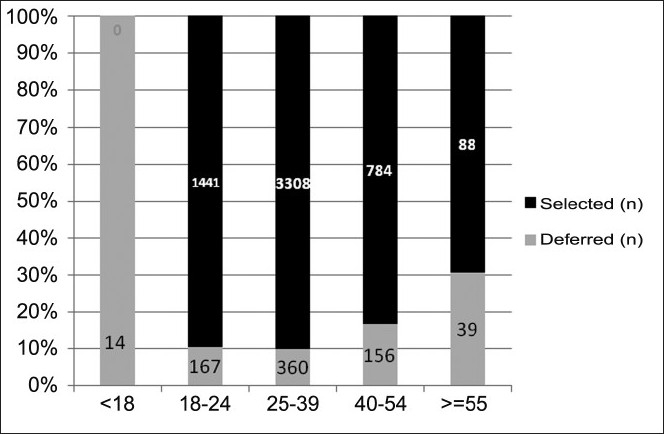
Percentage deferral in different age groups (age in years).

Deferral percentage increased significantly (*P*<0.05) as the age of the donor increased to greater than 40 years [Table 3] and more donors were deferred due to abnormal B.P. readings with increasing age [Table 7 and Figure 3].

There was no significant difference (*P*=0.57) in mean Hb values of male and female donors deferred due to low Hb (Table 8; Hb values in gm/dl).

**Table 8:** Mean Hb values for male and female donors deferred due to low Hb.

Gender	Mean	N	Std. Dev.	Minimum	Maximum	Median
Female	11.175	264	1.0472	6.9	12.4	11.5
Male	11.108	147	1.2837	5.6	12.4	11.4
Total	11.152	411	1.1343	5.6	12.4	11.4

A total of 82 donors were deferred due to abnormal B.P. readings on repeated testing on the day of presentation for donation [Tables 4 and 5]. Out of this, 76 were deferred for high and 6 for low systolic and/or diastolic readings. There was no significant difference in mean systolic B.P. values for deferred and selected donors (*P*>0.05), whereas a significant difference was found in mean diastolic blood pressure values between two groups (*P*<0.05), which was mainly due to wide standard deviation in deferred donors.

## Discussion

Blood donor suitability criteria based on science, informed medical opinion, and regulatory rules influence donor demographics and lead to specific deferral patterns.[5]

These criteria are designed to protect both the blood donor and the recipient from harm. The donor selection process results in deferral or rejection of potential blood donors who may not particularly like this feeling of being rejected and thus avoid returning for future donations.[2,5] Donor deferral rates in blood centers vary from 5 to 24%[6] leading to huge losses in terms of available blood units for transfusion in the nation every year.

We undertook this retrospective study to obtain the incidence of deferral in our whole blood donors and to analyze the deferral pattern with an aim to review our recruitment and retention strategy.

Past studies have segregated deferred donors on the basis of duration of deferral (i.e. temporary/short term or permanent)[3,7] and deferral due to pathological or non-pathological causes.[4] In our study, we segregated donor deferrals on the basis of medical interview or physical examination. Our objective was to formulate definite strategy based on “point of exit” of prospective blood donor in order to increase the efficiency of the donor screening process.

Deferral incidence in our study was 11.6% which was similar to that observed by Zou *et al*,[8] (12.8%) Chaudhary *et al*,[4] (16.4%), Bahadur and colleagues[3] (9%) and Custer *et al*,[7](13.6%). However, studies by other authors have cited low[9,10] (5.6-7.1%) to very high[6,11–13] (20-35.6%) deferral incidence in their donor populations, which probably reflects the regional diversity[12] and marked variation in whole blood donor eligibility criteria internationally.[14]

Knowledge of deferral incidence and causes in a particular region helps in deciding the magnitude and direction of blood donor recruitment efforts. This knowledge also helps in calculating the eligible and potential blood donor pool. The eligible donor pool may drastically vary from the potential donor pool which is usually calculated on the basis of age alone (population between 18 and 60 years of age). This fact was highlighted by William Riley and colleagues in their study where they showed that the conventional method of determining eligible donors, using age alone as the criteria, overestimated eligible donor prevalence (calculated using deferral incidence) by approximately 59 percent![15] Thus deferral data need to be calculated for various regions and the entire country to guide the recruitment efforts of blood collection services. State blood transfusion councils and regional blood centers can act as nodal centers for collecting and collating such data for the region which can then be merged at national level to obtain the complete picture.

Like most of other studies done in the past,[3,7,8,9,13,10,16] the most common reason for deferral in our whole blood donor population was low hemoglobin and nearly 56% of total deferrals were because of this. Nearly two third of these anemic donors were females highlighting the prevailing anemia in general population among females. Also, odds ratio indicated very high probability of deferral in females as compared to males due mainly to low Hb. There was no difference in deferral in voluntary and replacement donors due to low Hb.

Efforts are needed to address the issue of anemia in prospective blood donors at the regional, state, and national level and efforts are warranted on the lines of National Anemia Action Council (NAAC), Blood Center of Wisconsin.[17] NAAC showed that health of blood donors can be improved by educating and motivating them to seek medical attention for anemia, thus improving the eligibility of prospective blood donors in the long run. Simultaneously, association can be established with programs currently running in India for alleviating the problem of iron deficiency anemia like the “Twelve by Twelve Initiative”[18] targeting anemia eradication during adolescence. Again, the nodal bodies/centers can take the initiative for coordinating such programs whereby most common cause of deferral---a low Hb, can be tackled in blood donors.

The second and third most common reasons for deferral found in our study were abnormal blood pressure (almost all due to high B.P.) and donor on medication warranting deferral, respectively. Except for low hemoglobin, studies have found different common reasons for deferral in blood donors reflecting on the variation in donor population and eligibility criteria used in different parts of the world. Kwa *et al*,[10] (poor vein and underweight donors), Charles *et al*,[11] (low Hb and Hypertension), Zou *et al*,[8] (travel to malaria area and miscellaneous blood exposure), and Rabeya *et al*,[9] (high blood pressure and medical illness) have cited various other common reasons for deferral in respective study population.

In India, Bahadur and colleagues[3] in their study with predominantly replacement donors (99.4%) found low Hb as the most common cause of deferral (32.9%). However, second and third most common reasons in their study were low weight (26.6%) and history of jaundice/ hepatitis (8.1%). Similarly in another Indian study by Chaudhary and colleagues[4] low weight (32.3%) and low Hb (18.6%) were respectively the two most common reasons for the deferral.

This difference in the common reasons for deferral in various studies could have been because of varying proportion of replacement (almost all) as well as female donors (very low) in these studies, as compared to high female donor proportion (10%) and predominantly voluntary donors in our study (70.7%). This is supported by the fact that in our study too, the third most common cause of deferral in related donors was low weight while it was history of medication in voluntary donors. Additionally, use of electronic B.P. equipments, with more objective readings, in our study could have picked more hypertensive donors. However, this presumed difference in deferral reasons between replacement and voluntary donor needs to be validated by further studies.

Hypertension can lead to deferral of a significant percentage of prospective blood donors as evident in our study and another by Bahadur *et al*.[3] However, any blood donor suffering from a marked degree of hypertension has to be bled with care as in such cases the sudden removal of 350 or 450 ml of blood may precipitate a cerebral catastrophe.[19] This could be tragic for the donor as well as blood center bleeding such donor. Thus we need to find a “cut-off” after careful studies in our population so that we do not lose donors either ways. Available cut off needs to be validated in the light of recent studies as more and more donors can be presumed to be reaching this cut-off soon.

Our study found that the related donors had a significantly low deferral rate as compared to voluntary donors (as mentioned earlier, the related donors in our study were not replacement” donors as they were under no obligation to donate). Although reason for this difference was not clear, it can be presumed that there is a tendency of “self deferral” in related donors and only those who feel more comfortable or have higher awareness of donation process present themselves for blood donation. This presumption is supported by the overall lesser percentage of deferrals in related donors (22.3%) in medical interview as compared to voluntary donors (31.6). Also, significantly fewer related donors gave a positive history of medication in days immediately prior to presenting for donation [Table 5]. Thus knowledge about the possibility of deferral and awareness of deferral criteria may be responsible for lower deferral rate in related donors. Similar observation were made by Zou *et al*, in their study in the North American population where they showed that travel deferrals for vCJD risk saw a gradual fall due to self deferral and general awareness of these criteria in the public.[8,20]

However, contrary to our hypothesis, in the study done by Charles *et al*, in Trinidad and Tobago,[11] the difference in rate of deferral amongst voluntary and replacement donors was not significant. Demographics of the study population could be one important reason for this lack of difference in deferral rate, as is evident from a very high overall deferral rate in both voluntary and replacement donors (31.7% and 35.4%, respectively). Also, most common reason for deferral was high risk sexual activity in their study, unlike low Hb found in our study.

Nonetheless, it can be safely said that more studies on larger number of donors are needed to further test the impact of knowledge of deferral criteria in prospective blood donors. Theoretically speaking, IEC material providing information and education on causes and duration of deferral may “prime” prospective donors about possibility of deferral. Any such sensitization beforehand results in better acceptability of “rejection” and thereby less “negative” feeling about blood donation and more chances of future return. Dorothy *et al*,[21] supported this view that medical examination may actually serve as an incentive for future repeat donations. All selected or deferred donors who are given an explanation, feel motivated to check their eligibility to donate blood and return for future donation.

Increasing age was found to be a significant variable in our study, leading to increasing deferral of prospective blood donors. Again, hypertension was one of the common reasons for this increasing deferral in “senior” donors. Recruitment strategies thus need to take into consideration high deferral rate in elderly donors especially when camps are planned at places with higher proportion of donors above 40 years of age.

## Conclusion

Criteria for whole blood donor selection and deferral in India are based partially on scientific facts ““borrowed”” from developed countries and partially on tradition. However, sufficient “in-house” data and its scientific validation are still required to test the applicability of these criteria in our blood donors. Deferred donors can be considered somewhere in-between the chain of “an unsensitized donor---first time donor---regular donor” so far as recruitment strategies are concerned. They are better than uninitiated prospective donors but a little “behind” the regular repeat donors. Also, they are aware of the donation process and have at least once shown the willingness to donate. Salient findings in our study on predominantly voluntary blood donors are as follows.

- Incidence of deferral was 11.6%.
- Most common reasons for deferral were low Hb (55.8%), abnormal blood pressure (11.1%), and history of antibiotic/medication use (6.9%).
- Related donors had significantly lower deferral rate (5.80%) as compared to voluntary donors (13.97%).
- Chance of deferral in female blood donors is nearly 15 times higher as compared to male donors.
- Increasing age was associated with higher chances of deferral in prospective blood donors.
- This study had limited power owing to the small sample size; however these findings still provide pointers to the blood-banking community for future action.
- Determine incidence of donor deferral at state and national level.
- Educate, motivate, and treat donors deferred due to anemia/low Hb, so that they can be recruited again.
- Rationalize and revalidate deferral criteria based on studies done in Indian population; for example criteria for Hb cutoff and criteria for acceptable B.P.
- Create awareness (using IEC material) about deferral criteria, statistics, and duration among prospective blood donors.
- Modify recruitment strategy according to locally and regionally prevalent donor demographics.

Effective measures thus need to be initiated to address the issue of lost donors in terms of “how much” and “why.” It is high time to take stock of the present and future precious blood units lost due to these deferrals. Existing channels of data collection for blood donations in the country can be restructured to mark the beginning in this direction.
